# Impact of the T296S mutation in P450 GcoA for aryl-O-demethylation: a QM/MM study

**DOI:** 10.3389/fchem.2023.1327398

**Published:** 2024-01-11

**Authors:** Sonia F. G. Santos, Rajesh Reddy Bommareddy, Gary W. Black, Warispreet Singh

**Affiliations:** ^1^ Hub for Biotechnology in Build Environment, Newcastle upon Tyne, United Kingdom; ^2^ Department of Applied Sciences, Northumbria University, Newcastle upon Tyne, United Kingdom

**Keywords:** catalysis, aryl-O-demethylation, Cytochrome P450, molecular dynamics, QM/MM

## Abstract

Lignin, a complex plant cell wall component, holds promise as a renewable aromatic carbon feedstock. p-Vanillin is a key product of lignin depolymerization and a precursor of protocatechuic acid (PCA) that has tremendous potential for biofuel production. While the GcoAB enzyme, native to *Amycolatopsis* sp., naturally catalyzes aryl-O-demethylation toward guaiacol, recent research introduced a single mutation, T296S, into the GcoA_P450_ enzyme, enabling it to catalyze aryl-O-demethylation of p-vanillin. This structural modification increases the efficiency of GcoA_P450_ for the natural substrate while being active for p-vanillin. This study reveals the increased flexibility of p-vanillin and its ability to adapt a favorable conformation by aligning the methoxy group in close proximity to Fe(IV) = O of Cpd I in the active site of the T296S variant. The QM/MM calculations in accordance with the experimental data validated that the rate-limiting step for the oxidation of p-vanillin is hydrogen atom abstraction and provided a detailed geometric structure of stationary and saddle points for the oxidation of p-vanillin.

## 1 Introduction

**FIGURE 1:**
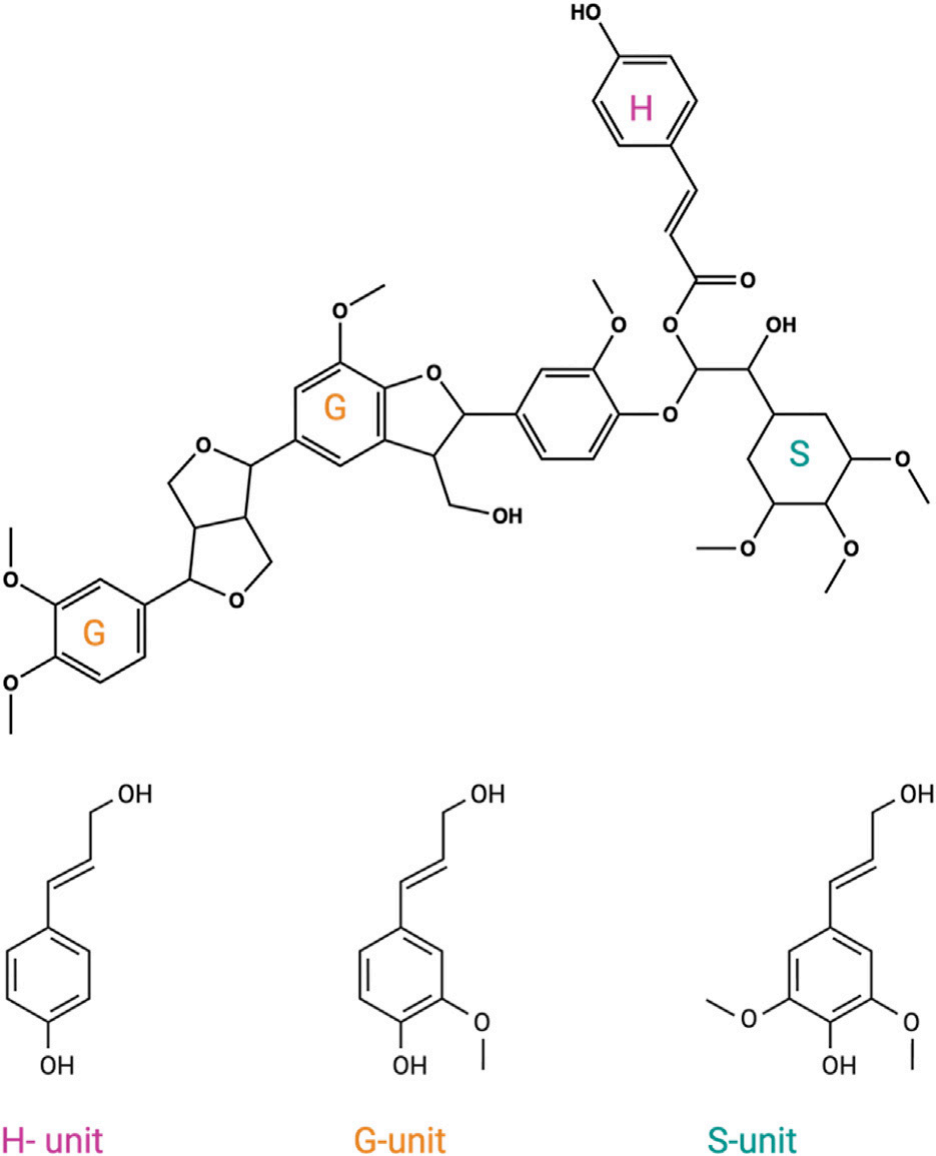
Representation of the three aromatic monomers of lignin biopolymer: coniferyl alcohol (G-unit) (orange), sinapyl alcohol (S-unit) (green), and p-coumaryl alcohol (H-unit) (pink).

Lignin, a complex aromatic biopolymer found predominantly in plant cell walls, plays multiple vital roles, such as providing structural support, defending against pathogens, and facilitating water and nutrient transport within plant tissues (Ralph et al., 2004; Ragauskas et al., 2014; Li and Takkellapati, 2018; Mallinson et al., 2018; Machovina et al., 2019; Ellis et al., 2021; Singh et al., 2022). It is primarily composed of three aromatic building blocks: coniferyl alcohol (G-unit), sinapyl alcohol (S-unit), and p-coumaryl alcohol (H-unit) (Boerjan et al., 2003; Vanholme et al., 2010). Lignin stands out as a valuable and renewable source of aromatic carbon feedstock (Figure 1). Lignocellulosic biomass typically consists of cellulose, hemicellulose, and lignin. However, owing to its recalcitrance, lignin cannot be utilized by conventional fermentation, which accounts for up to 40% of lignocellulosic biomass (Sun and Cheng, 2002). One promising approach for harnessing its potential lies in the microbial conversion of these aromatic compounds (Guengerich and Macdonald, 1990; Mallinson et al., 2018; Ponnusamy et al., 2019; Ali et al., 2020; Davaritouchaee et al., 2020; Fetherolf et al., 2020; Ellis et al., 2021; Singh et al., 2022; Santos et al., 2023). A crucial and rate-limiting step in this process involves the aryl-O-demethylation of methoxy groups within lignin monomers, leading to the formation of diols (Mallinson et al., 2018; Machovina et al., 2019; Ellis et al., 2021; Singh et al., 2022). This transformation sets the stage for subsequent oxidative aromatic ring-opening reactions. In a recent study, researchers reported their successful engineering of cytochrome GcoAB_P450_. This achievement hinged on a single mutation, T296S, which endowed the enzyme with the capability to demethylate p-vanillin, a crucial depolymerization product from lignin (Ellis et al., 2021).

**FIGURE 2:**
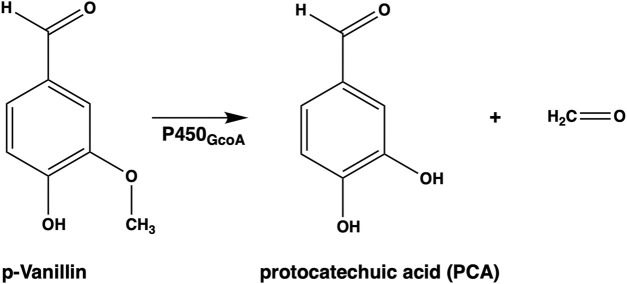
Schematic representation of the aryl-O-demethylation of p-vanillin performed by GcoA_P450_ producing protocatechuic acid and a formaldehyde molecule.

The GcoAB enzyme was isolated from the bacterial species *Amycolatopsis* sp. Its classification places it within the cytochrome P450 family as CYP255A, and it was found to catalyze aryl-O-demethylation toward guaiacol (Mallinson et al., 2018; Machovina et al., 2019; Ellis et al., 2021). This aryl-O-demethylation reaction holds immense significance in the lignin breakdown process and the subsequent metabolization of its products (Mallinson et al., 2018; Machovina et al., 2019; Singh et al., 2022). The aryl-O-demethylation of p-vanillin results in the production of protocatechuic acid (PCA), an antioxidant with high electroactivity, which is a potential sustainable and low-cost fuel (Figure 2) (Homma et al., 2021). The discovery of an enzyme capable of this reaction, particularly when its natural substrate is guaiacol, a G-unit of the lignin biopolymer, piqued the interest of researchers (Ellis et al., 2021). However, in the case of p-vanillin, a highly abundant product of lignin depolymerization, GcoA_P450_ exhibited little to no activity (Mallinson et al., 2018; Ellis et al., 2021). To address this limitation, Ellis et al. (2021) embarked on a journey of structure-guided protein engineering, coupled with biochemical assays and molecular dynamics (MD) simulations, to gain a better understanding of the mutations and their impact on enzyme efficacy. Their efforts led to the identification of a promising group of mutations for catalyzing aryl-O-demethylation. Notably, they reported that a single mutation, T296S, transformed GcoA_P450_ into an efficient biocatalyst capable of turning over the p-isomer of vanillin. Remarkably, this variant retained substantial activity against the native GcoA substrate, guaiacol. The enhanced reaction efficiency appeared to stem from GcoA_T296S_’s ability to form productively oriented complexes with p-vanillin (Ellis et al., 2021).

The structural analysis of the T296S variant revealed that this small modification created sufficient space to accommodate the aldehyde of p-vanillin and that the residue S296 is able to stabilize CpdI, consequently mitigating the shift observed in the heme propionate group closest to residue T296 when in complex with p-vanillin. The residue 296, as a serine, has the flexibility and ability to stabilize both the substrate and the Cpd I. This variant closely resembled the GcoA_WT_–guaiacol complex, restoring both the water environment and the original heme position (Ellis et al., 2021).

The T296S single-point mutation improving the catalytic activity of GcoA_P450_ left questions about how this mutation affected the reaction mechanism unanswered. This study’s primary objective is to decipher why the T296S mutant can demethylate p-vanillin while the wild-type enzyme has little to no activity against the same substrate. To address these differences, we used molecular dynamics (MD) and quantum mechanics/molecular mechanics (QM/MM) calculations for both the wild-type and mutant forms.

## 2 Methods

We used MD simulations for conformational analysis and hybrid QM/MM calculations for reaction kinetics. The detailed protocols are stated below.

### 2.1 Structure preparation

For the initial structure of the native GcoA_P450_ enzyme in complex with vanillin, we used the crystal with PDB ID 5OMR (Mallinson et al., 2018) and used USCF chimera (Pettersen et al., 2004) to mutate T296S. The protonation states of the side chains of the titratable amino acids were assessed using the H++ server at pH 7.5, which corroborates well with previous literature (Mallinson et al., 2018; Ellis et al., 2021). The force field parameters for the Cpd I were taken from the previous literature (Shahrokh et al., 2012). The force field parameters for p-vanillin were developed using the general Amber force field (GAFF) using the Antechamber module of the Amber package (Wang et al., 2001). The partial charges of these compounds were calculated with the restrained electrostatic potential (RESP) method of a QM-optimized geometry using the Gaussian 16 package (Frisch et al., 2016) at the HF/6-31G* level of theory. We used the Amber ff14SB force field for protein molecules and the TIP3P water model for the solvents. Missing hydrogen atoms and an appropriate number of counterions to neutralize the complexes were added by the leap module of Amber 20 (Case et al., 2020). Thereafter, each complex was immersed into a truncated octahedral box of TIP3P water molecules, with the boundary of the protein system being 15 Å away from the box edges. The periodic boundary conditions were used in all the simulations. Long-range electrostatic interactions were calculated using the particle mesh Ewald (PME) with a cut-off of 12 Å for the direct space Coulomb and van der Waals forces.

### 2.2 MD simulations

All simulations were performed using the GPU version of PMEMD integrated with Amber 20 (Case et al., 2020). The solute molecules were restrained using a potential of 5 kcal mol^−1^ Å^2^, and the solvent and ions were subjected to energy minimization (5,000 steps) using the steepest descent and conjugate gradient methods. The entire system was then subjected to controlled heating from 0 to 298.15 K for 50 ps at constant volume using a Langevin thermostat with a collision frequency of 1 ps^−1^ using a canonical ensemble. During the heating process, non-hydrogen atoms of solute molecules were restrained using a harmonic potential of 5 kcal mol^−1^ Å^2^. This was followed by another round of energy minimization for 2,000 steps using the steepest descent and conjugate gradient methods. The entire system was then subjected to two rounds of equilibration at 298.15 K for 50 ps using a weak restrain of 0.1 kcal mol^−1^ Å^2^ on all the solute atoms in an NPT ensemble. A Berendsen barostat was used to maintain the pressure at 1 bar, and the SHAKE algorithm (Miyamoto and Kollman, 1992) was used to constrain bonds involving hydrogen. A time step of 2 fs was used for all MD runs. A production MD run for continuous 14 subsystems for the GcoA_WT_ complex and the GcoA_T296S_ variant was performed in an NPT ensemble with a target pressure of 1 bar with a pressure coupling constant of 2 ps for each system, which gave us an overall sampling of 500 ns. The data were saved for every 50 ps. The root mean square deviation (RMSD), distances between residues, hydrogen bonding, radial distribution function, and cluster analysis were conducted using the combination of cpptraj (Roe and Cheatham, 2013) and pytraj using Jupyter Notebook (Kluyver et al., 2016).

### 2.3 QM/MM calculations

The reaction profiles of GcoA_P450_ wild type and the recombinant in complex with p-vanillin were studied using QM/MM calculations implemented in ChemShell (Sherwood et al., 2003). The QM calculations were performed using ORCA 4.2.0 (Neese, 2012), and the MM part was defined using DL_POLY (Smith and Forester, 1996). The effect of the protein environment on the polarization of the QM wavefunction was described by the electronic embedding scheme. Snapshots for the QM/MM calculations were obtained from the equilibrated MD trajectory using cluster analysis. The representative snapshots from cluster analysis were then subsequently subjected to energy minimization using the steepest descend (1,250) and conjugate gradient (1,250) algorithms using Amber 20 (Case et al., 2020). The water shell within 4 Å of the protein or within 20 Å of the QM atoms was retained. The QM region consists of the whole Cpd I molecule, ligands, and the cysteine residue truncated at Cβ positions. The residues within 10 Å of Cpd I and ligands, including water molecules, were allowed to move freely, and the rest of the system was frozen during geometry optimization. The hydrogen link atoms were used to saturate the dangling bond at the QM/MM boundary. The reaction coordinate was defined by the distance between the oxygen atom of the Fe(IV) = O in Cpd I and the hydrogen atom to be abstracted. The transition state (TS) structure connecting the reactant and the product was obtained by performing a relaxed potential energy surface (PES) scan with an increment of 0.1 Å. All QM calculations were performed with DFT using UB3LYP (Lee et al., 1988; Miehlich et al., 1989) with D3 dispersion correction and BJ damping (Grimme et al., 2011), with the def2-SVP basis in the doublet state. The RIJCOSX (Grimme et al., 2011) approximation was used in QM calculations. Frequency calculations were carried out for ZPE and validating the transition states obtained. Only one unique imaginary frequency was present, with the normal mode of the imaginary frequency corresponding to the transition of the hydrogen atom from the C–H bond to the oxygen atom of the Fe(IV) = O of the Cpd I. All the QM/MM calculations of GcoA_T296S_ and GcoA_WT_ started from CpdI as it is the main oxidant in P450-mediated reactions (Singh et al., 2022).

## 3 Results and discussion

### 3.1 T296S structural impact on the active site

Fourteen replicas of 500 ns MD simulations were conducted for both the native GcoA_P450_ and GcoA_T296S_ in complex with p-vanillin. This provided us with an overall sampling of 14 μs to study how T296S mutation enhances the aryl-O-demethylation of p-vanillin to PCA. The stability of the native GcoA_P450_, GcoA_T296S_, and p-vanillin ligand were verified via RMSD analysis, as shown in Supplementary Figures S1–S4. Additionally, the MD simulations consistently showed a stable orientation of the substrate’s methoxy group toward the oxo-iron complex, as shown in Supplementary Figures S5, S6.

**FIGURE 3:**
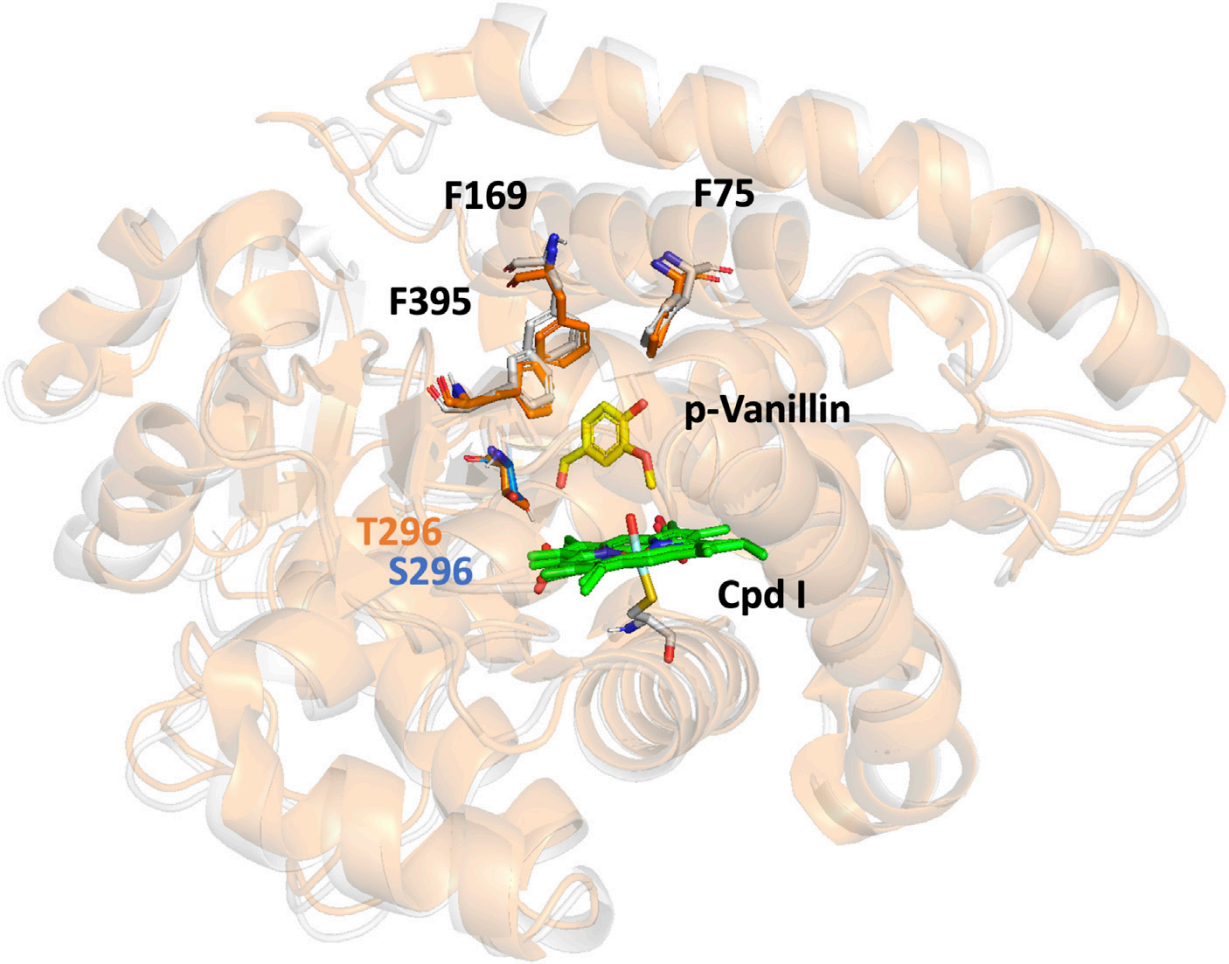
Representation of the GcoA_WT_ (orange) and the recombinant GcoA_T296S_ (gray) superimposed and showing closed conformation. The substrate p-vanillin (yellow licorice) is in the active center of the enzyme as the CpdI (green licorice). The aromatic triad consisting of F75, F169, and F395 is represented in both enzymes. The residue T296 (orange licorice) and the mutation S296 (blue licorice) are represented, and their position relative to the substrate is clear.

Upon analyzing the complexes, it became evident that the closed conformation observed in the X-ray crystal structure is consistently maintained throughout the MD simulations. Furthermore, the aromatic triad consisting of F75, F169, and F395 remains in a stable conformation (as shown in Figure 3), effectively serving as gatekeepers to control water access into the active site pocket, in accordance with prior studies (Mallinson et al., 2018; Singh et al., 2022).

Although the GcoA_P450_ enzyme demonstrates the capability to conduct aryl-O-demethylation of guaiacol, a representative of the G-unit lignin monomer, it exhibits minimal to no activity when interacting with p-vanillin, a significant product derived from lignin depolymerization (Mallinson et al., 2018; Ellis et al., 2021). However, through a single mutation, T296S, the enzyme becomes highly productive while retaining its catalytic efficiency toward guaiacol, its natural substrate (Ellis et al., 2021). In this study, we conducted an analysis of the positioning of p-vanillin within the active sites of both native and recombinant GcoA_P450_.

**FIGURE 4:**
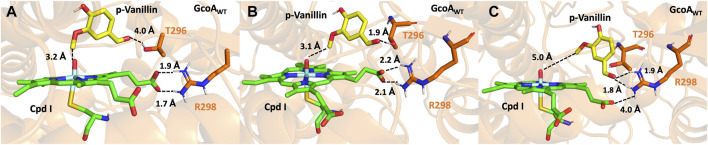
Representation of the most populated conformations during the MD simulation of GcoA_WT_ in complex with p-vanillin. **(A)** This active site conformation is productive, and from this reactant, the aryl-O-demethylation can indeed happen. However, as shown in **(B)** and **(C)**, the bond formed between the T296 and the aldehyde group of the substrate pulls the substrate, breaking the bonds between R298 and the Cpd I, leaving it unstable, and the substrate is in a non-productive pose ending in a non-active complex.

In the presence of guaiacol, the native protein forms bonds between two residues (T296 and R298) with the propionate chain of compound I to establish a stabilizing interaction (see Supplementary Figure S7). However, upon the entry of p-vanillin into the active site of GcoA_WT_, structural adjustments occur due to the extra aldehyde group compared to guaiacol. This leads to a disruption in the stabilization of compound I, causing T296 to shift, and the hydroxy group of the residue moves toward the aldehyde group of the substrate (Figure 4A). Additionally, the bond between R298 and the oxygen in the propionate chain is severed (Figure 4C).

During the molecular dynamics (MD) simulation, it is observed that the substrate is drawn toward nearby residues, assuming a non-productive conformation (Figures 4B, C). This phenomenon potentially results in decoupling, contributing to the notably low catalytic efficacy of GcoA_WT_ toward p-vanillin.

However, when the reactant assumes the correct conformation (at the beginning of the MD simulations), as shown in Figure 4A, the energy barrier for the rate-limiting step of HAA closely resembles that of GcoA_T296S_ (see Supplementary Figure S8 and Supplementary Table S1). This similarity accounts for the modest yet observable O-demethylation of GcoA toward p-vanillin.

**FIGURE 5:**
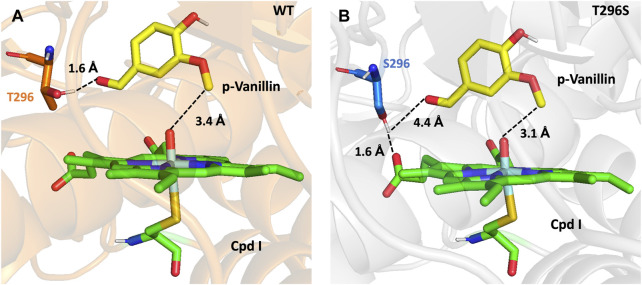
**(A)** Representation of the substrate p-vanillin (yellow licorice) in the active center of the GcoA_WT_ (orange cartoon) showing a bond of 1.6Å between the T296 (orange licorice) and the aldehyde group of the substrate and with the demethyl group by 3.4 Å from the oxo-iron complex (green licorice). **(B)** Representation of the substrate p-vanillin (yellow licorice) in the active center of GcoA_T296S_ (gray cartoon) showing a distance of 4.4 Å between the S296 (blue licorice) and the aldehyde group of the substrate, and with the demethyl group by 3.1Å from the oxo-iron complex (green licorice). The distances are shown in Å.

Substituting T296 with serine imparts flexibility to the residue, enabling it to alternate between two different conformations either stabilizing the aldehyde group of the substrate or propionate side chain of CpdI. This results in the stabilization of both entities in an optimal and productive conformation, as shown in Figure 5. This representation mirrors the original environment, particularly in the vicinity of the propionate chain of CpdI, similar to when guaiacol is bonded (refer to Supplementary Figure S7).

### 3.2 Mechanism of aryl-O-demethylation in GcoA_T296S_ for p-vanillin

As shown in the previous section, the substrate p-vanillin has an extra aldehyde group that does not exist in guaiacol, the natural substrate of GcoA_P450_. When in the active site of GcoA_WT_, T296 makes a hydrogen bond to the aldehyde group of the substrate, breaking the interaction with the propionate side chain of CpdI. Therefore, replacing that residue by a serine, the substrate is free to move and adopt the best conformation for catalysis and the mutated residue moves towards the propionate chain stabilizing the Cpd I. The conformation adopted by p-vanillin is the same that guaiacol adopts in GcoA_WT_, and the environment surrounding the Cpd I is also restored by this mutation.

**FIGURE 6:**
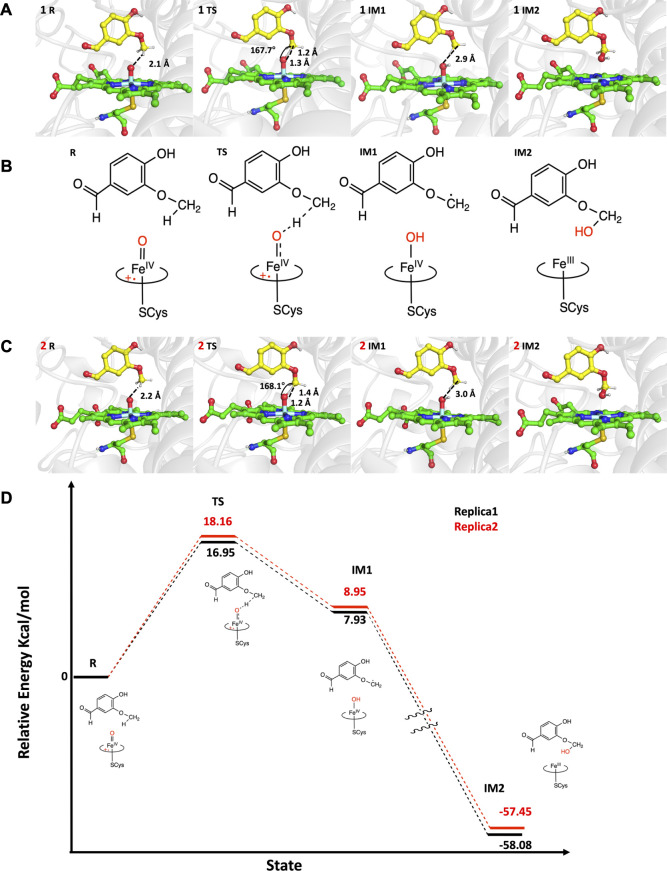
QM/MM reaction profile of HAA and the following rebound step performed by Cpd I. **(A)** Representation of each step of the aryl-O-demethylation mechanism on replica 1 performed by the Cpd I (green licorice) of GcoA_T296S_ in complex with p-vanillin (yellow) was computed using the UB3LYP functional with D3BJ dispersion correction and the def2-TZVP basis set for S = ½. **(B)** Representation of aryl-O-demethylation step by step. **(C)** Representation of each step of the aryl-O-demethylation mechanism on replica 2 performed by the Cpd I (green licorice) of GcoA_T296S_ in complex with p-vanillin (yellow) was computed using the UB3LYP functional with D3BJ dispersion correction and the def2-SVP basis set for S = ½. **(D)** QM/MM reaction profile of replica 1 (black) and replica 2 (red). The key distances are shown in Å.

To gain a deeper understanding of the catalytic activity facilitated by the T296S mutation in GcoA_P450_ and its influence on the reaction mechanism (Figure 6B), we conducted QM/MM calculations of two replicates using snapshots derived from the most frequently occurring structure obtained from cluster analysis of the equilibrated MD trajectory. Figure 6 shows the reaction profiles for replica 1 (Figure 6A and Supplementary Table S2) and replica 2 (Figure 6C) in black and red, respectively (Figure 6D).

In both replicas, aryl-O-demethylation is initiated by hydrogen atom abstraction (HAA) from the methoxy group of p-vanillin by Cpd I, the main oxidant in cytochrome P450 enzymes. In replicas 1 and 2, the substrate is in a vertical position, and the hydrogen to be abstracted in the methoxy group of p-vanillin is located approximately 2.1 and 2.2 Å, respectively, from the oxo group of Cpd I. The transition state (TS) associated with HAA also shows very similar distances and angles for both replicas 1 and 2 and goes through an activation free energy of 17.0 kcal/mol and 18.2 kcal/mol, respectively. This geometric structure of the TS is consistent with our previous work on the oxidation of guaiacol, syringol, and 4-propylguaiacol by GcoA_WT_ and its close homolog AgcA_P450_ (Singh et al., 2022; Santos et al., 2023). In both replicas, the intermediate IM1 is endothermic in nature and has a slight difference in energy, with 7.93 kcal/mol for replica 1 and 8.95 kcal/mol for replica 2. These results are consistent with the previous work where the IM1 was also endothermic in nature (Singh et al., 2022; Santos et al., 2023). The next step in the reaction profile which is a common feature in P450 chemistry is the transfer of the hydroxyl group, also known as the rebound step, from the iron oxo complex to the methoxy group of the substrate. This is a barrierless process and results in the formation of an exothermic hydroxylated product (IM2) with a final energy of −58.08 kcal/mol for replica 1 and -57.45 kcal/mol for replica 2. The IM2 leaves the active site and is demethylated non-enzymatically in solution. A similar process of demethylation was studied by Shaik et al. in an aqueous solution (Wang et al., 2015). Our findings also validate that HAA indeed represents the rate-limiting step for aryl-O-demethylation, a conclusion further substantiated by experimental data (Ellis et al., 2021), where recombinant GcoA_P450_ T296S exhibited a Kcat of 0.63 s^−1^, which corresponds to an activation free energy of 16.01 kcal mol^−1^.

## 4 Conclusion

To unlock the potential of microbial conversion of lignin’s aromatic compounds, which serve as a plentiful and promising source of aromatics, the initial crucial step involves depolymerizing lignin. This process yields various compounds, including methoxylated compounds, such as the p-isomer of vanillin. This aldehyde exhibits great potential in the biofuel domain as it serves as a precursor to protocatechuic acid (PCA), a potential sustainable and cost-effective fuel source. However, this transformation relies on aryl-O-demethylation, a catalytic reaction that can be achievable through essential P450 biocatalysts.

The introduction of the T296S mutation in the GcoA_P450_ enzyme had significant structural implications. Our MD simulations, in accordance with the previous work (Ellis et al., 2021), show that the T296S mutation disrupted the robust hydrogen bond between the side chain of T296 and the aldehyde group of the substrate p-vanillin. Consequently, p-vanillin shows flexibility in the active site of the GcoA_T296S_ variant and is no longer constrained by the hydrogen bond as it did in the GcoA_WT_ enzyme. In the active site of the GcoA_T296S_ variant, p-vanillin adopts a favorable conformation by aligning the methoxy group in close proximity to Fe(IV) = O of Cpd I. The MD simulations also revealed that the binding conformation of p-vanillin in the T296S GcoA_P450_ variant is very similar to the X-ray structure binding mode of guaiacol in the wild-type GcoA_P450_. Beyond that, S296 forms a hydrogen bond with the propionate chain of Cpd I and participates in its stabilization, restoring the natural surrounding environment of CpdI.

The QM/MM calculations in accordance with the experimental data validated that the rate-limiting step for the oxidation of p-vanillin is hydrogen atom abstraction and provided the detailed geometric structure of stationary and saddle points for the oxidation of p-vanillin. GcoA_P450_ has gained the ability to carry out the crucial aryl-O-demethylation reaction not only for its native substrate, guaiacol, but also for p-vanillin, a significant product generated during lignin depolymerization. This expanded capability holds promise in the field of biofuels.

## Data Availability

The original contributions presented in the study are included in the article/Supplementary Material; further inquiries can be directed to the corresponding author.
