# Supplementary material for: Impact of the T296S mutation in P450 GcoA for aryl-O-demethylation: a QM/MM study
Source: Front Chem. 2024 Jan 11;11:1327398. doi: 10.3389/fchem.2023.1327398 (PMC10811788; doi:10.3389/fchem.2023.1327398)
Supplement: Supplementary file 1 [file DataSheet1.docx]

Supplementary Material

# Supplementary Figures and Tables

## Supplementary Figures


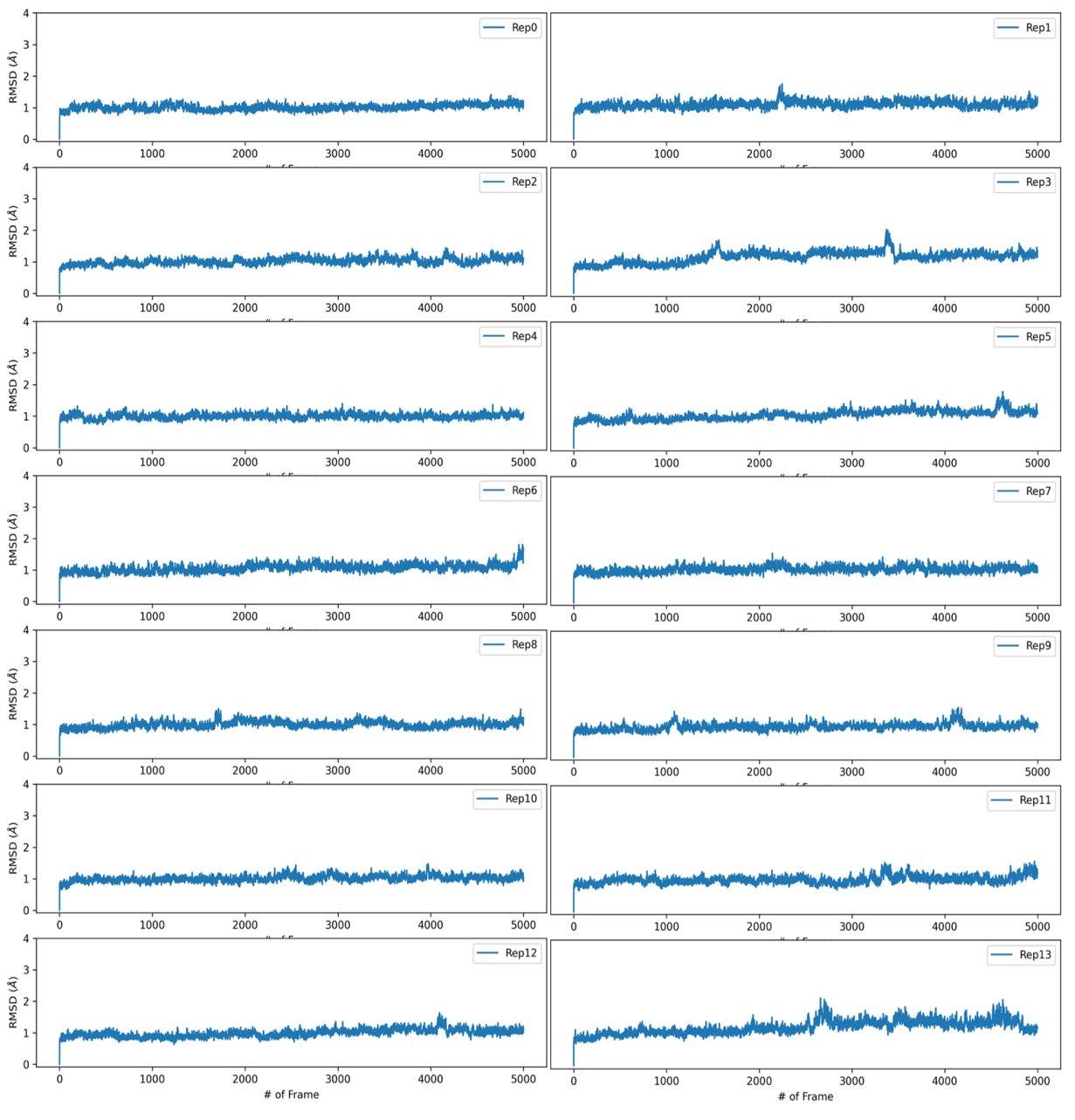


Supplementary Figure 1. RMSD for the backbone Cα atoms of GcoA_WT_.


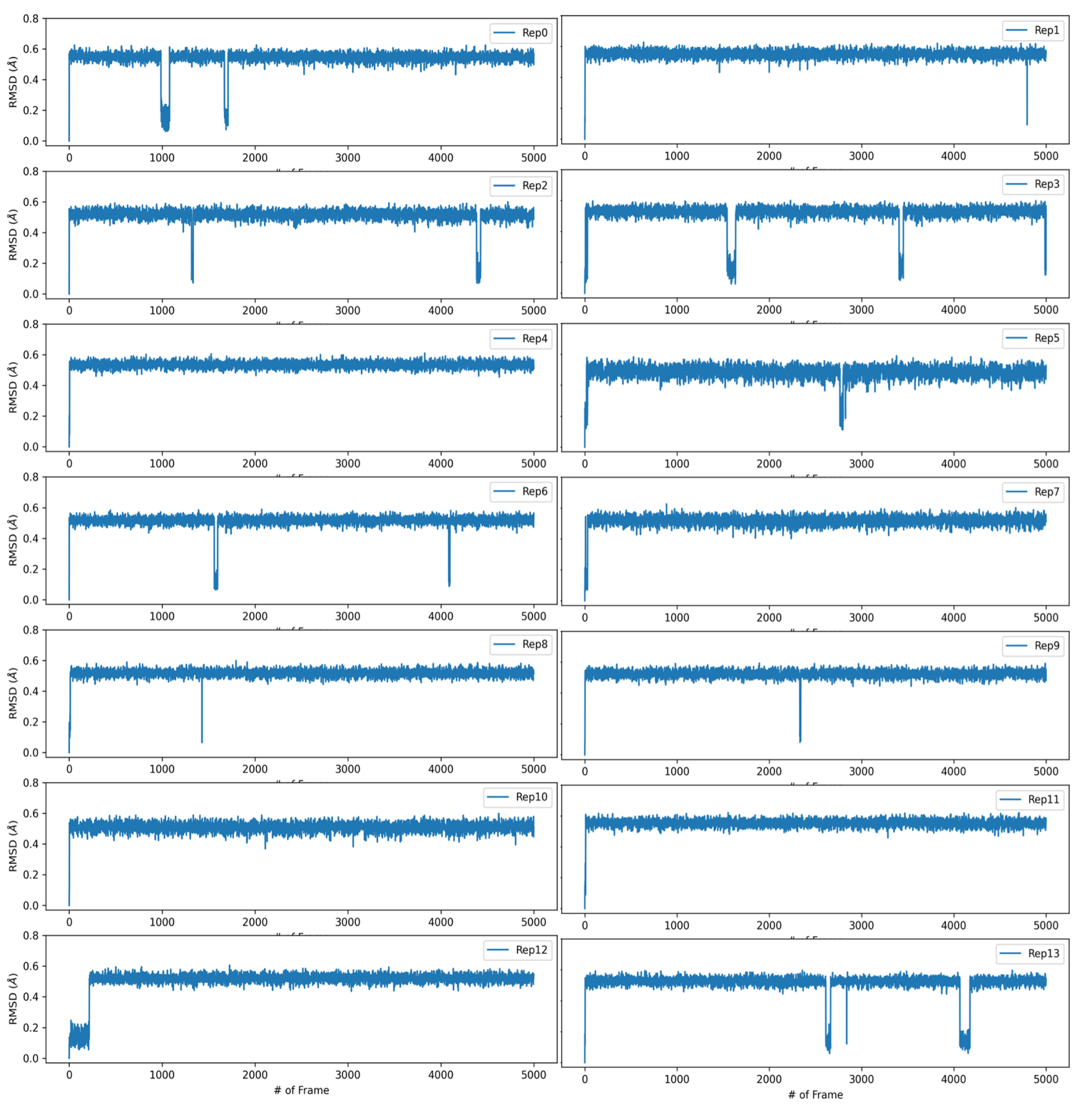


Supplementary Figure 2. RMSD of the heavy atoms of substrate p-Vanillin in complex with the GcoA_WT_.


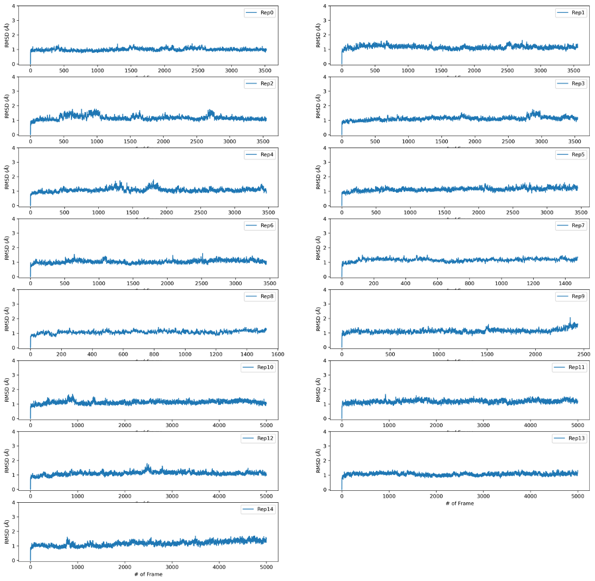


Supplementary Figure 3. RMSD for the backbone Cα atoms of recombinant GcoA_T296S_ protein.


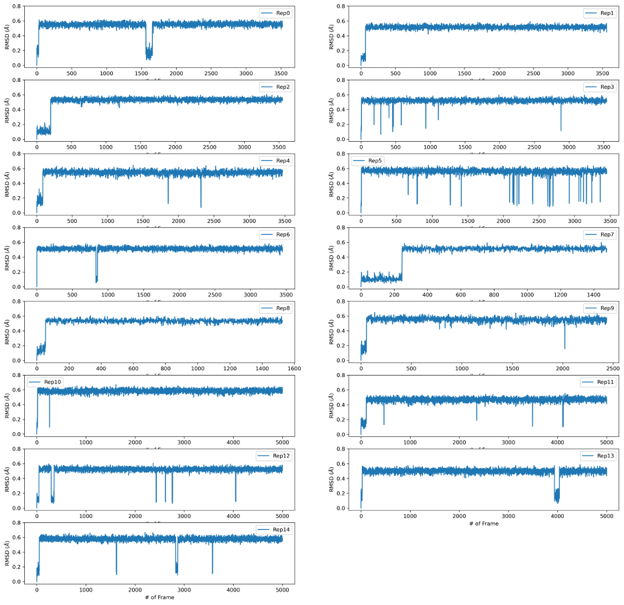


Supplementary Figure 4. RMSD of the heavy atoms of substrate p-Vanillin in complex with the recombinant GcoA_T296S_.


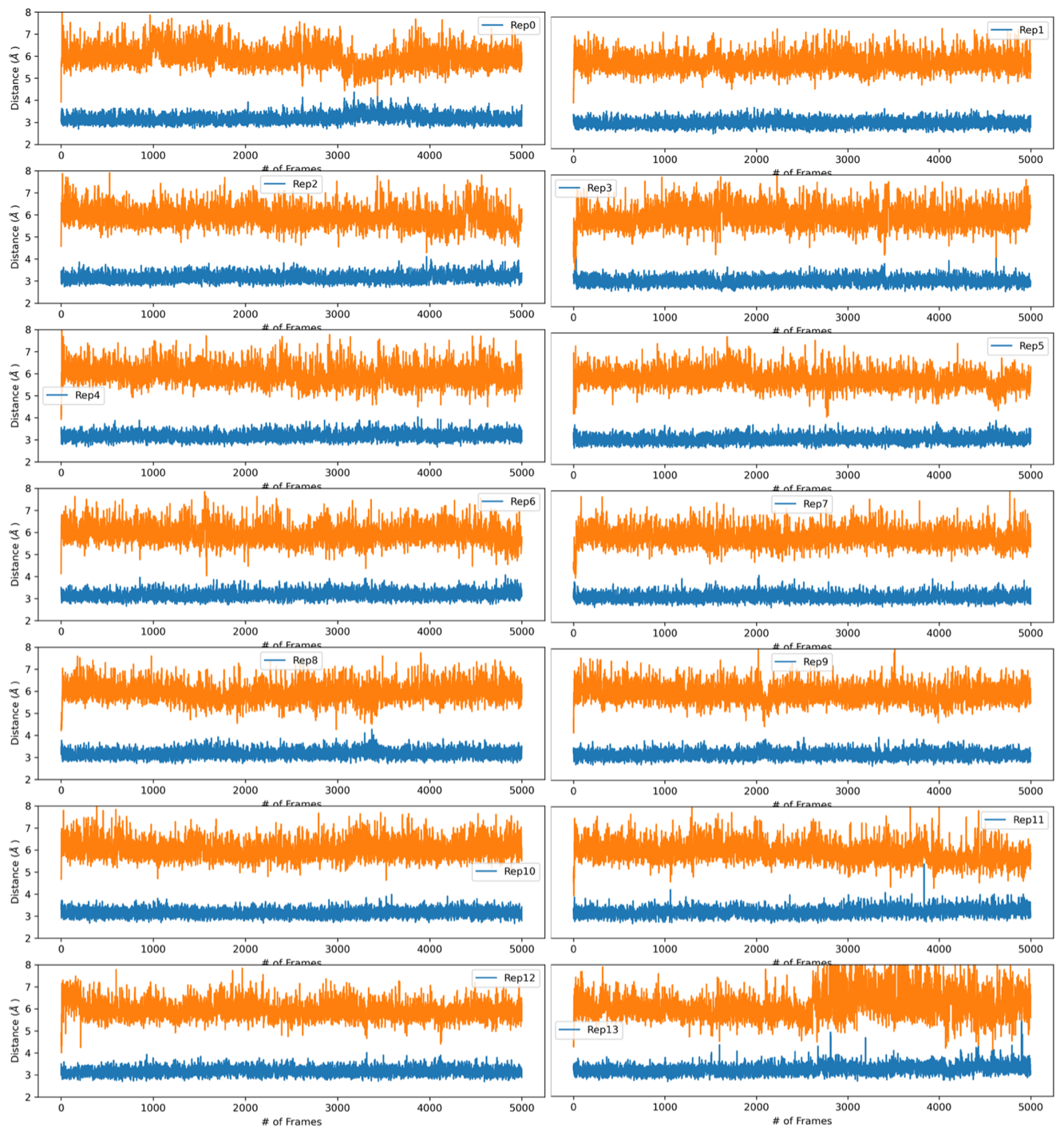


Supplementary Figure 5. Distance between the oxygen atom of the substrate’s aldehyde group and the oxo-iron complex (orange) and the distance of the substrate’s methoxy group and the oxo-iron complex (blue). The distances were obtained from the MD simulations of GcoA_WT_ in complex with p-Vanillin.


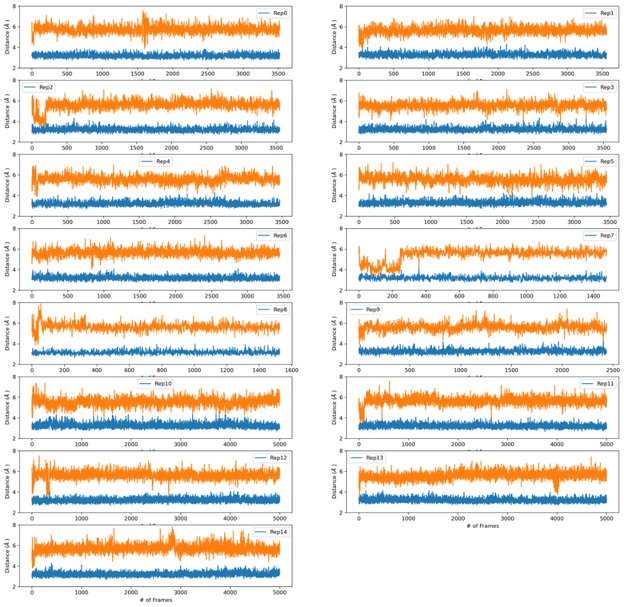


Supplementary Figure 6. Distance between the oxygen atom of the substrate’s aldehyde group and the oxo-iron complex (orange) and the distance of the substrate’s methoxy group and the oxo-iron complex (blue). The distances were obtained from the MD simulations of GcoA_T296S_ in complex with p-Vanillin.


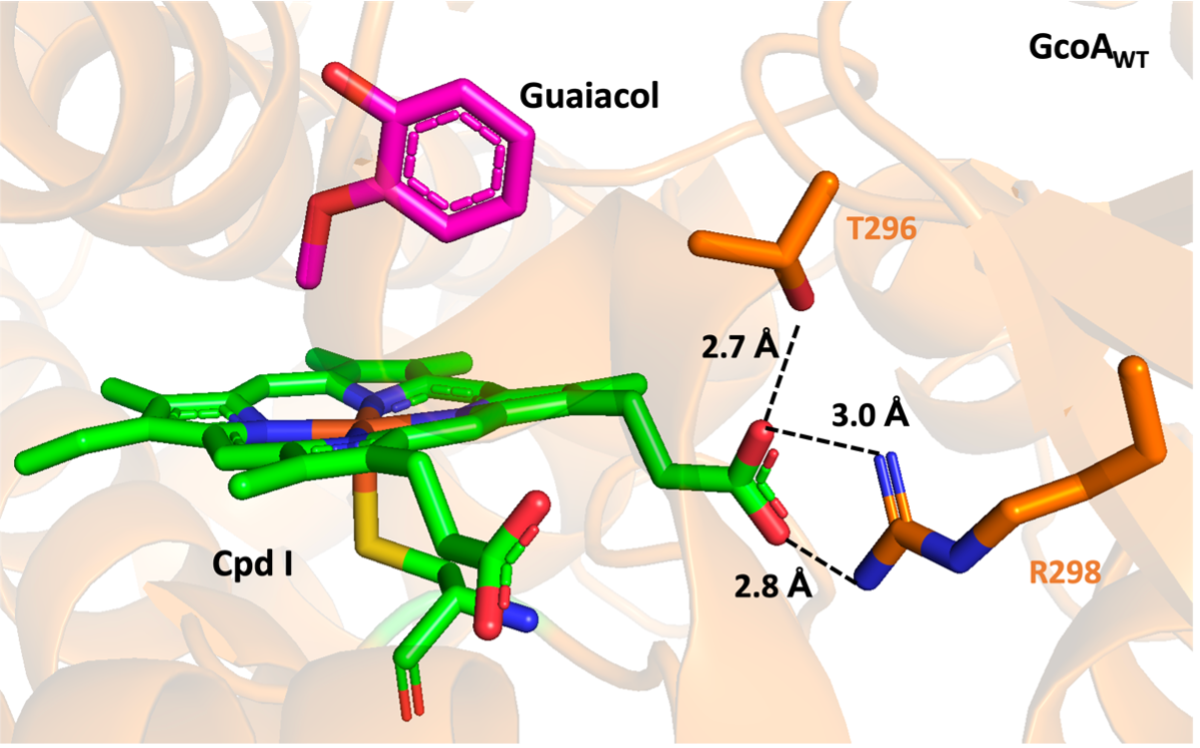


Supplementary Figure 7. Representation of the protein GcoA_WT_ in complex with its natural substrate, Guaiacol. Here we can see that the Propionate chain of CpdI is stabilized not only by the ARG298 but also for THR296.


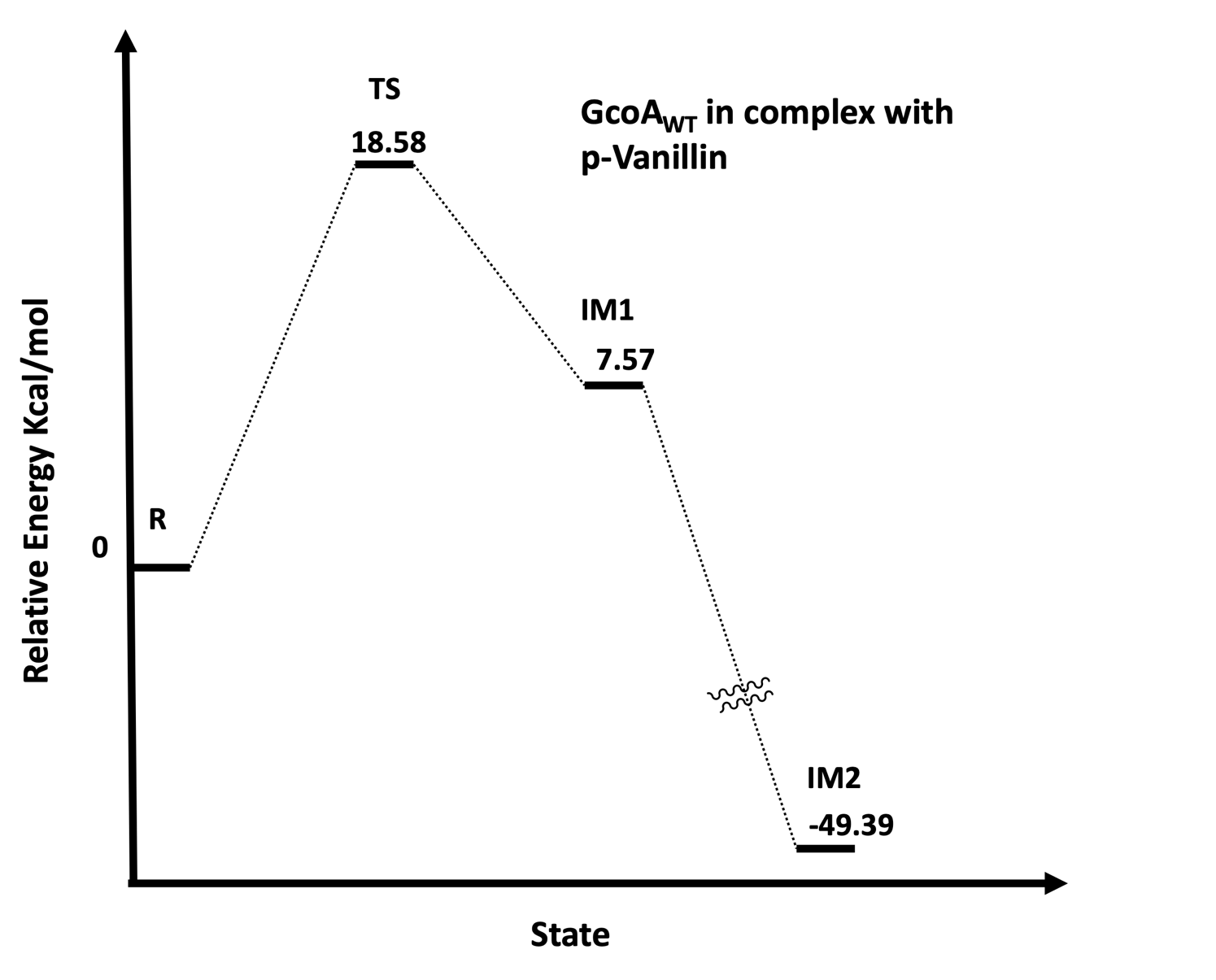


Supplementary Figure 8. QM/MM reaction profile of GcoA_WT_ in complex with p-Vanillin.

## Supplementary Tables

Supplementary Table 1. QM/MM calculations at M=2 of GcoA_WT_ in complex with p-Vanillin. The reaction profile started from the best productive conformations for the hydrogen atom transfer (HAT) catalysed by Cpd I was computed using the UB3LYP functional with D3BJ dispersion correction and with Zero-point energy corrections.

|  | **E** | **ZPE** | **E+ZPE** | **E kcal/mol** | **E +ZPE kcal/mol** |
| --- | --- | --- | --- | --- | --- |
| **R** | -4222.972438 | 0.758784547 | -4222.213654 | 0 | 0 |
| **TS** | -4222.936221 | 0.75216902 | -4222.184052 | 22.72651461 | 18.57521194 |
| **IM1** | -4222.957186 | 0.755599536 | -4222.201587 | 9.570892619 | 7.572269551 |
| **IM2** | -4223.054037 | 0.761676861 | -4222.29236 | -51.20380732 | -49.38885463 |

Supplementary Table 2. QM/MM calculations at M=2 of Replica 1 of GcoA_T296S_ in complex with p-vanillin. The reaction profile of the hydrogen atom transfer (HAT) catalysed by Cpd I was computed using the UB3LYP functional with D3BJ dispersion correction and with Zero-point energy corrections.

|  | **E** | **ZPE** | **E+ZPE** | **E kcal/mol** | **E +ZPE kcal/mol** |
| --- | --- | --- | --- | --- | --- |
| **R** | -4223.580489 | 0.758525299 | -4222.821964 | 0 | 0 |
| **TS** | -4223.546173 | 0.751217286 | -4222.794955 | 21.53367773 | 16.94783349 |
| **IM1** | -4223.564665 | 0.755341613 | -4222.809323 | 9.929558484 | 7.931767054 |
| **IM2** | -4223.677806 | 0.763279648 | -4222.914526 | -61.06722841 | -58.08383174 |
